# Dynamic fine-tuning of CAR-T cell therapy

**DOI:** 10.1016/j.omto.2023.06.001

**Published:** 2023-06-29

**Authors:** Pierre V.M. Trehin, Geisler Muñoz-Guamuro, Wilfried Weber

**Affiliations:** 1 INM – Leibniz Institute for New Materials, Campus D2 2, 66123 Saarbrücken, Germany; 2 Department of Materials Science and Engineering, Saarland University, Campus D2 2, 66123 Saarbrücken, Germany; 3 Signalling Research Centres BIOSS and CIBSS and Faculty of Biology, University of Freiburg, Schänzlestrasse 18, 79104 Freiburg, Germany

Cancer immunotherapy has transformed cancer treatment, with chimeric antigen receptor (CAR)-T cell therapy being one of the most promising approaches. In the previous issue of *Molecular Therapy – Oncolytics*, Lainšček et al.^1^ outline a novel strategy for controlling CD19 CAR-T cell activity to address limitations currently hampering clinical practice. To better and dynamically align CAR-T cell activity with clinical needs, the authors developed a method for external control based on engineered endogenous transcription factors acting downstream of the CAR signaling pathway.

CAR-T cells are T cells that have been genetically modified to express CARs on their surface, allowing them to recognize and target specific tumor antigens. This approach has shown overwhelming success in treating hematological malignancies such as acute lymphoblastic leukemia and non-Hodgkin’s lymphoma.^2^^,^^3^ However, significant challenges remain to be addressed to improve safety and efficacy. This is mainly linked to the fact that the activity of the engineered cells, once in the patient, remains difficult to control: insufficient activation of CAR-T cells yields inefficient tumor killing, whereas uncontrolled CAR-T cell activation can result in serious complications such as cytokine release syndrome (CRS), neurotoxicity, and organ damage.^4^ CRS is a systemic inflammatory response caused by the release of large amounts of cytokines into the bloodstream by activated CAR-T cells.

**Figure 1:**
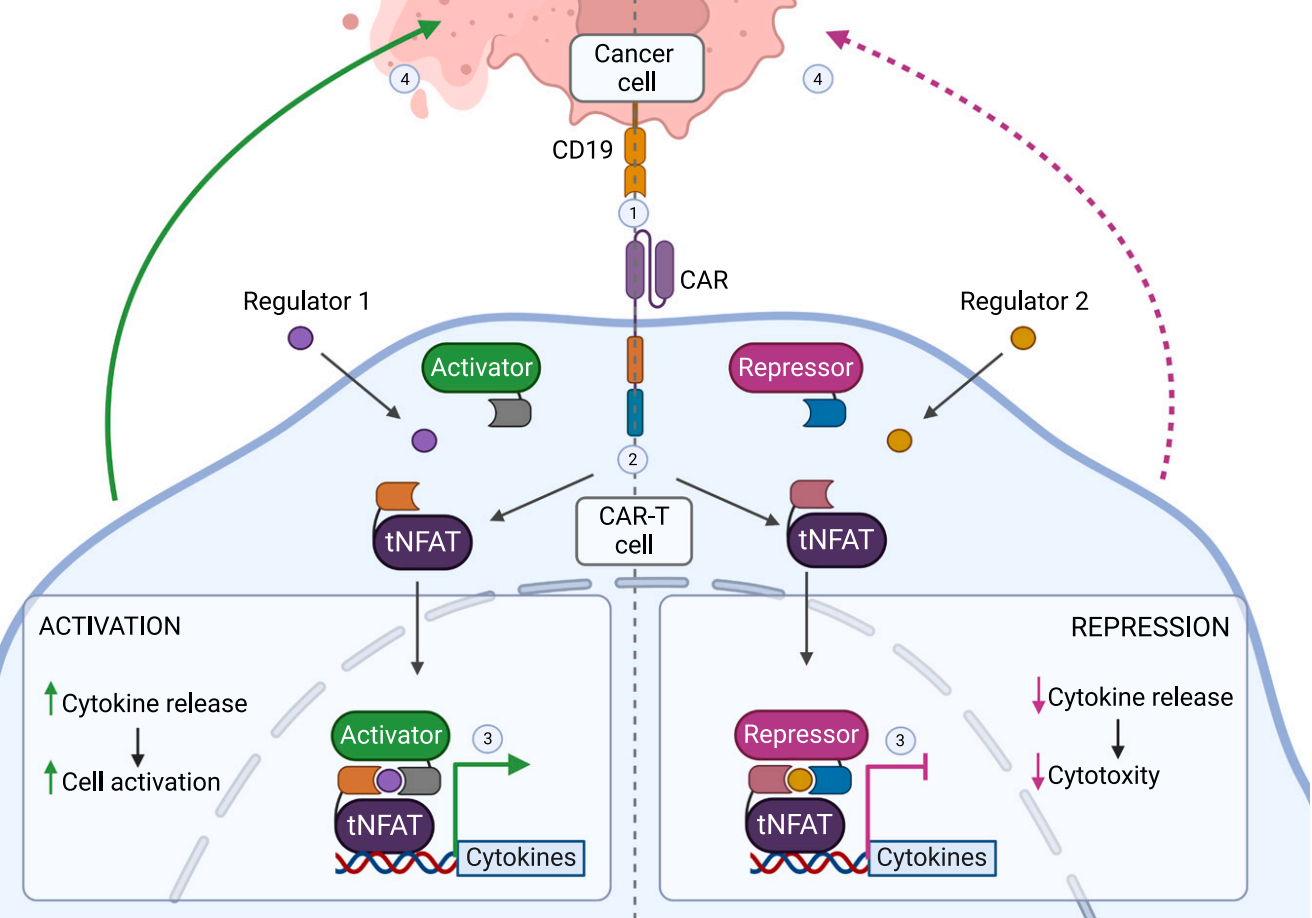
Dynamic control of CAR-T cell activity based on an engineered TF To achieve dynamic control over CAR-T cell activity, Lainšček et al. fused tNFAT to binding domains of inducible HDs. The corresponding protein-binding partners of HDs were fused to transcriptional activator or repressor domains. Thus, the recruitment of an activator or repressor, to enhance or reduce the activity of CAR-T cells respectively, was regulated by the external addition of small chemical compounds (regulators).

To achieve dynamic control over CAR-T cell activity, Lainšček et al. selected the nuclear factor of activated T cell 2 (NFAT), truncated and fused it to binding domains of heterodimerization systems (HDs), whose activity can be regulated by the external addition of a small chemical compound. The corresponding protein-binding partners of HDs were fused to transcription activator or repressor domains. Thus, the externally controlled recruitment of either an activator or a repressor can be used to either increase or down-tune CAR-T cell activity, respectively (Figure 1). By lowering off-target effects and boosting tumor-specific targeting, the approach of Lainšček et al. may increase the safety and effectiveness of CAR-T cell treatment.

The authors fused the truncated NFAT (tNFAT) to the binding domains of three different inducible HDs: abscisic acid, gibberellin, and rapamycin. The interaction counterpart of the HDs was fused to transcriptional activator (VPR) or repressor (KRAB) domains to create NFAT transcription factors (TFs) that can influence gene transcription. The activation of the engineered TFs was successfully shown in HEK293 cells, where they significantly upregulated or downregulated the activity of an NFAT-specific synthetic promoter driving the expression of a firefly luciferase (fLUC) reporter gene. The potency of the regulation system was further confirmed in Jurkat T cells expressing a CD19 CAR construct, where the systems were able to significantly upregulate and downregulate IL-2 cytokine secretion. Maximal regulation was observed for the abscisic acid-induced HD system. To further improve the system and allow a reversible activation, the tNFAT was fused with different HDs at each of its ends, enabling it to link to both the activator and the repressor, depending on the stimulation. It showed a promising amount of reversibility, especially for OFF-ON-OFF cycles where the abscisic acid-activator and gibberellin-repressor system showed strong IL-2 expression upon induction of the activator system and a large decrease in secretion upon the induction of the repression system.

When used with human T cells from healthy donors, retrovirally transduced with CD19 CAR, the best performing abscisic acid-based system shows a precise upregulation or downregulation of both IL-2 and IFN-γ expression when in contact with CD19^+^ BCWM cancer cells derived from a patient with B cell lymphoma. Importantly, in this case, cancer cell killing was observed upon induction, which shows that the system influences not only cytokines production, but also overall T cell functionality. For *in vivo* validation, mice were inoculated with BCWM-fLUC xenograft cancer and were treated with CD19 CAR-T cells transduced with the abscisic acid-activator system. In contrast with CD19 CAR-T cells without the engineered TF-based system, cells activated by abscisic acid showed significant immunotherapeutic properties as measured by bioluminescence imaging loss from the fLUC cancer cells. Mice with the activated system also showed a lower final tumor burden, and their survival rate was significantly higher than other groups. However, the influence of activation on T cell proliferation remains to be investigated.

The potential for reversibility allows this technique to compare favorably to technologies like inducible Casp9,^5^ which allows for the rapid removal of CAR-T cells at the onset of CRS via a suicide gene, as the level of control which it gives access to could allow for an attenuation of the symptoms while continuing the therapy instead of stopping it outright. The level of control of this technique also enables the targeted regulation of CAR-T cells, whereas systemic inhibitors like Dasatinib allow for fast inhibition of all T cells of the patient.^6^ Additionally, controlling the activity of CAR-T cells at the NFAT level grants wide control over T cell activity, including cancer cell killing and CAR-T cell proliferation. This is not the case for approaches that concentrate on the transcription of the CAR, such as CAR Tet-On systems.^7^ Finally, comparable levels of specificity are reached by Boolean logic gate systems using AND and NOT gates. Especially NOT gates using inhibitory CAR allow a high level of discrimination between healthy and tumor tissue and restrict the proliferation of CAR-T cells in tumor tissue.^8^ This is especially important for treating solid cancer, in models of which NFAT showed increased cancer killing but the regulation technology was not tested yet.

The abscisic acid-regulated NFAT system may, however, be limited by the non-human origin of the chemically-inducible dimerizarion system, which may trigger an immune response, an issue that should be further tested *in vivo*. The PYL1 protein for instance could elicit an immune response, as has been seen against other non-human protein components used as regulators, such as viral-derived Ns3a proteases or HSV-TK. Some of these systems have also been used for immunotherapeutic control over CAR-T cells.^9^^,^^10^ Computational prediction and targeted mutation of protein-protein interacting domains that can serve as major histocompatibility complex-presenting epitopes can aid in lowering the potential immunogenicity of non-human derived regulators. The testing having been performed in immunocompromised mice does not yet answer these questions. Results in more physiologically relevant systems using additional cancer models will give more information concerning important parameters of the technology, including the stability and availability of the activating chemicals.

Overall, this article showcases a promising technology addressing important bottlenecks in current CAR-T therapy. It will be interesting to see how this approach may further develop in future preclinical trials.
